# Gender influences end-of-life decisions

**DOI:** 10.1186/cc9942

**Published:** 2011-03-11

**Authors:** ME Lissauer, L Smitz-Naranjo, SB Johnson

**Affiliations:** 1 RA Cowley Shock Trauma Center, Baltimore, MD, USA

## Introduction

End-of-life care is an unavoidable component of critical care. Despite palliative care guidelines, wide variations exist in patient selection and implementation of limitations in care decisions. Understanding why some patients have care limited and some are provided full resuscitative efforts allows opportunities for improving care at the end of life.

## Methods

All consecutive deaths (*n *= 151 patients) in a tertiary-care surgical ICU over a 2.2-year period were reviewed. Patients were divided into groups: withhold (WH) = patients who had potentially life-saving therapies withheld/withdrawn; full care (FC) = patients who had full resuscitative efforts prior to death. Demographics, acute physiology score (APS), and APACHE IV scores were used to compare groups. Fisher's exact test and Student's *t *test (significance: *P *< 0.05 level) were used.

## Results

A total of 1,764 patients were admitted and 151 (8.6%) died. Patients who died had a mean age of 63 ± 14 years and 83 (55%) were male. One hundred and eleven (74%) had potentially life-saving therapy withheld/withdrawn (WH group). Forty patients (26%) had full resuscitative efforts until time of death (FC group). Age, admission APACHE IV, and APACHE IV at time of death/withdrawal of care were similar between genders, however significantly more males had care withdrawn than females (83% vs. 47%, *P *< 0.005). Compared with the FC group, the WH group was less sick at ICU admission (APS: 76.7 ± 28.3 vs. 91.7 ± 37.0, *P *< 0.01) but had similar pre-existing co-morbidities (chronic health points: 13.3 ± 7.2 vs. 11.7 ± 6.9). Compared with their admission APS, both groups had similar deteriorations in clinical status and the FC group remained significantly more ill (APS 93.6 ± 31.4 vs. 109.4 ± 44.7, *P *< 0.02 between groups and *P *< 0.05 compared with admission). Factors not different between groups included: APACHE diagnosis, admitting service, admitting source (ED, OR, floor, other hospital), need for mechanical ventilation, or readmissions. Specifically there were no differences between groups in types of chronic illnesses including cancer, liver disease, COPD, diabetes or in ICU length of stay (18 ± 17 vs. 16 ± 37).

## Conclusions

Gender more than age, severity of illness, diagnosis, and co-morbidities had a profound influence on end-of-life care and decisions. Duration of the ICU stay and deteriorating status did not appear to impact decisions to limit care. The FC group was more sick at ICU admission and at time of death than the WH group. Gender issues at end of life need to be further studied to optimize limitations of care for all patients.

